# 

**Published:** 1880-07

**Authors:** 


*CONTENTS.*

| | Page |
|---|---|
| Consumption | 181 |
| Heart Disease | 184 |
| Still They Come | 185 |
| Cold Bathing | 187 |
| Drinking at Meals | 189 |
| To Bring Up Children | 191 |
| Health of Employments | 193 |
| Politics and Physic | 195 |
| Throat-Ail Causes | 198 |
| To be Safe | 198 |
| Failing Eyesight | 199 |
| Mental Rest | 200 |
| The First Wedding | 203 |
| Sunshine | 204 |
| Premature Deaths | 207 |
| Success in Life | 207 |
| Bowel Complaints of Children | 209 |

